# Early Cochlear Implant Promotes Global Development in Children with Severe-to-Profound Hearing Loss

**DOI:** 10.3390/audiolres15050121

**Published:** 2025-09-22

**Authors:** Chiara Falzone, Letizia Guerzoni, Sara Ghiselli, Laura Franchomme, Maria Nicastri, Patrizia Mancini, Enrico Fabrizi, Domenico Cuda

**Affiliations:** 1 Department of Otorhinolaryngology, “Guglielmo da Saliceto” Hospital, Via Cantone del Cristo 40, 29121 Piacenza, Italy; c.falzone@ausl.pc.it (C.F.); l.guerzoni@ausl.pc.it (L.G.); s.ghiselli@ausl.pc.it (S.G.); l.franchomme@ausl.pc.it (L.F.); 2 Department of Sense Organs, Sapienza University, 00184 Rome, Italy; maria.nicastri@uniroma1.it (M.N.); p.mancini@uniroma1.it (P.M.); 3 Department of Economics and Social Sciences, Università Cattolica del S. Cuore, Via Emilia Parmense 84, 29122 Piacenza, Italy; enrico.fabrizi@unicatt.it; 4 Department of Medicine and Surgery, University of Parma, 43121 Parma, Italy

**Keywords:** global development, cochlear implant, children

## Abstract

**Background/Objectives:** The primary objective of the present study was to investigate early global development in children after one year of cochlear implant (CI) use. The secondary objective was to investigate the role of variables such as age at CI activation, gender, and parental schooling in early global development in children with a CI. **Methods:** The study sample included 24 subjects. All children were affected by severe-to-profound congenital bilateral sensorineural hearing loss (HL). The HL was diagnosed between 1 and 23 months of age (median 3 months) and participants underwent cochlear implant activation at 9–25 months (median 14 months). Participants were evaluated before CI surgery and after one year of CI use using the Italian version of the Griffiths III scales. **Results:** The general developmental quotient remained stable, as did the developmental quotients on scales A, C, D, and E. However, the development quotients on scale B, corresponding to the domain of “language and communication,” underwent a significant increase (*p* value < 0.05). There was a statistically significant negative effect of “age at CI activation” on both DQ at scale B (t − 3.457) and GDQ (t − 42.069). Maternal schooling had a significant positive effect on GDQ and DQ for scales A to D (*p*. value < 0.05). **Conclusions:** After one year of CI use, a significant improvement in the early global development of children was found in the language and communication domain. The age at CI activation and the level of the mother’s education were found to be related to early global development.

## 1. Introduction

Over the past decades, the use of cochlear implants (CIs) in children has significantly increased, mainly due to the widespread implementation of newborn hearing screening programs and advancements in pediatric audiology [1,2,3,4,5]. Early diagnosis and prompt cochlear implantation have been strongly associated with better language acquisition outcomes and improved auditory development [6,7].

Despite such evidence, there is still much variability in outcomes with cochlear implant (CIs) that could be attributable to factors such as age at implantation and diagnosis, as well as environmental factors like parental education and home language environment [8,9].

Cognitive abilities like attention, memory, and executive functions also seem to play a crucial but underexplored role in post-implantation outcomes compared to linguistic abilities, for example [10,11].

Measures of intelligence are the broadest and most valid indicators of developmental abilities in children and are significantly related to language learning, behavioral and social development, and school success [12,13].

Furthermore, most of them are focused on non-verbal cognitive ability measurements at school age [14,15,16].

For these reasons, there is a need for more comprehensive studies that evaluate global development at an early age in children with cochlear implants.

The primary objective of the present study was to investigate early global development in children after one year of CI use. The secondary objective was to investigate the role of variables such as age at CI activation, gender, and parental schooling in early global development in children with a CI.

## 2. Materials and Methods

### 2.1. Study Design

This is a prospective, observational, and nonrandomized study. All parents signed an informed consent form. This study was approved by the institutional local ethics committee Comitato Etico dell’Area Vasta Emilia Nord (AVEN) (Prot. N. 2021/0120510).

### 2.2. Sampling Criteria

The recruited subjects were required to meet the following criteria:

- Diagnosis of severetoprofound deafness by 24 months age;
- Age at activation of the CI being within 36 months;
- Absence of pathologies associated with deafness (e.g., severe hypotonia or neuromotor disorders);
- Hearing parents;
- Exposure to an oral communication habilitation program before and after implantation;
- No exposure to sign language;
- Signing of informed consent.

The exclusion criteria were as follows:

- Presence of associated pathologies and neuromotor delays;
- Non-Italian-speaking parents.

All subjects underwent evaluation during routine check-ups at the outpatient clinics.

### 2.3. Participants

The study sample included 24 subjects (12 males, 12 females) who were observed at the Complex Operative Unit of Otolaryngology at Piacenza Hospital and the Policlinico Umberto I in Rome. All children were affected by congenital bilateral sensorineural hearing loss (HL) with the unaided pure tone average (PTA) at the frequencies 500–1000–2000–4000 Hz, and higher than 85 dB HL on the CI side.

The HL was diagnosed between 1 and 23 months of age (median 3 months) and participants underwent cochlear implant activation at 9–25 months (median 14 months). All participants were fitted with bilateral hearing aids (HAs) prior to receiving their first CI and underwent oral rehabilitation. In thirteen cases, a bilateral CI was performed; in the remaining eleven, a unilateral CI was performed. These children continued using the HA on the contralateral ear (bimodal stimulation).

Participants were evaluated before CI surgery (t0) and after one year of CI use (t1). The mean age of the sample at t0 was 13.7 months and at t1 it was 24.4 months.

The etiology of hearing loss in the sample is distributed as follows: 15 children with genetic hearing loss (12 with the gjb2 gene mutation, 2 with usher syndrome, 1 with Waardenburg syndrome); 1 malformation of the inner ear (enlarged vestibular aqueduct); 1 case of meningitis; 2 children born prematurely; and 5 cases of unknown etiology.

All children wore their bilateral HAs at t0. At this time the mean aided PTA was 73.8 dB; after implantation, at t1, it was 29.3 dB. The average age of mothers in schooling was 15.2 years (range 8–18), while that of fathers was 14 years (range 8–20). Table 1 summarizes the sample characteristics.

### 2.4. Materials

The early global development of the children in this study was evaluated using the Italian version of the Griffiths III scales [17].

The upgraded version of the test (Griffiths development scales III) was used here.

These are scales standardized on a population of 841 typically developing children aged between 1 and 72 months 

This test has good psychometric qualities: good reliability (r > 0.96) and high content validity, construct validity, discriminant validity, and convergent validity (*p* < 0.05) [18].

The test was administered at two points: before CI surgery (t0) and after one year of CI activation (t1) by a psychologist and speech therapists properly trained for this task. The Griffiths III test was, in fact, administered by pediatricians, psychologists, neuropsychiatrists, and other professionals (e.g., speech therapists) with appropriate certification.

The test provides an overall measure of a child’s development and an individual profile of strengths across five areas:

- Foundations of Learning (scale A): Assesses critical aspects of learning during the early childhood years. These aspects include basic cognitive skills for learning, such as attention and processing speed; thinking skills, such as reasoning, organizing information, and planning solutions; different types of memory (working memory, visual memory, auditory memory, etc.); and playing skills.
- Language and Communication (scale B): Measures overall language development. This includes expressive language, receptive language, and use of language to communicate socially with others.
- Eye and Hand Coordination (scale C): Tests fine motor skills, manual dexterity, and visual perception skills.
- Personal–Social–Emotional (scale D): Measures constructs relating to the child’s developing sense of self and growing independence, interactions with others, plus many aspects of emotional development.
- Gross Motor (scale E): Assesses postural control, balance, and gross body coordination, among other abilities.

A raw score is calculated for each scale, which can be converted into four different standard scores: weighted score, developmental quotient (DQ) (it has a mean of 100 and standard deviation of 15, and its distribution approximates a normal curve), stanine, and percentile. The global development quotient (GDQ) is also usually calculated. It is derived from the combination of the scores of the different subscales. The GDQ provides a general indication of a child’s level of development compared to his or her peers. The Griffiths III test provides a comprehensive assessment of a child’s developmental level. It generates a detailed development profile across various domains, enabling precise determination of age-appropriateness and the presence of any developmental delay. For the purposes of this study, we will extract the DQ for each subscale and the GDQ.

### 2.5. Statistical Analysis

To compare scale scores at different times, we used a paired *t*-test. Normality was assessed using a Shapiro–Wilk test; non-parametric alternatives to the paired *t*-test were unnecessary. *p*. values were adjusted for multiplicity using a Benjamini–Hochberg correction. In the multivariate analysis, we used linear mixed models to compare scale scores measured at different times, including other possibly confounding covariates [19]. In this case the normality of residuals was checked again using a Shapiro–Wilk test. All computations were carried out using the R software (version 4.4.3). Specifically, for the estimation of linear mixed models, we used the lme4 package [20].

## 3. Results

Table 2 shows the evolution of early development quotients through CI use.

Both GDQ and DQ (at various scales) are displayed. Quotients were calculated at two distinct times: pre-implantation (t0) and post-implantation (t1).

**Figure 1:**
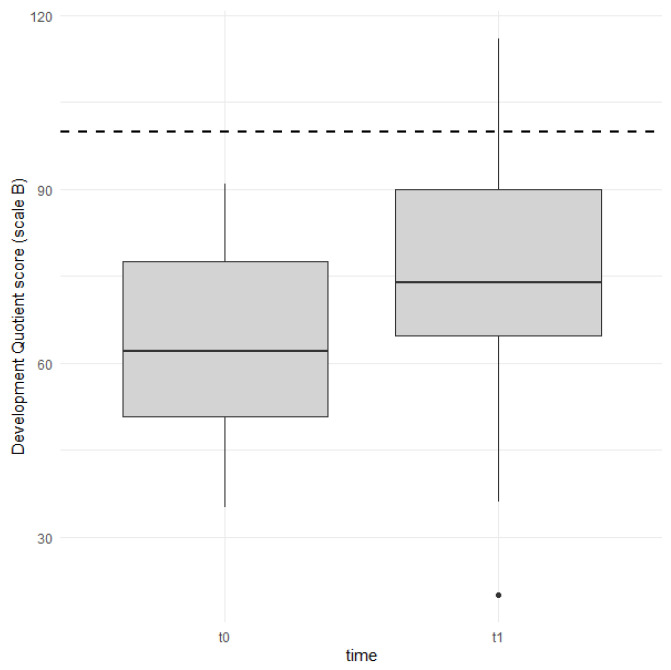
Box plots of DQs in scale B (“language and communication”) before (t0) and after 1 year of CI use (t1). Dotted line indicates reference normal value (DQ = 100).

The overall quotient (GDQ) remained stable, as did the DQs on scales A, C, D, and E. However, the development quotients on scale B, corresponding to the domain of “language and communication,” underwent a significant increase. Figure 1 shows this data in a box plot format, clearly displaying the development quotients for scale B. The differences between the pre- and post-implant values, along with the trend toward the reference lines, are both evident.

We achieved the second study’s objective with a multivariate analysis. We conducted this analysis for DQs at all scales and for the GDQ. We included the variables of age at CI activation, gender, and parental schooling.

Our data ruled out any substantial links between gender, father’s education, and early global development.

**Figure 2:**
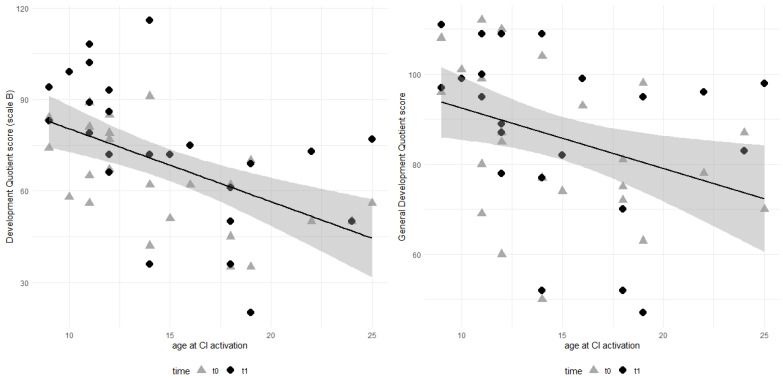
Distribution of scale B (“language and communication”) development quotients (**left panel**) and GDQ (**right panel**) at different ages at CI activation. A linear relationship is described by the black dark lines (shaded areas represent 95% confidence interval around the lines).

There was a statistically significant negative effect of “age at CI activation” on both the DQ at scale B (t − 3.457) and GDQ (t − 2.069). We checked the normality of residuals using a Shapiro–Wilk procedure, as these statistical tests rely on a normality assumption. We never rejected the null hypothesis. Figure 2 shows the scatterplot and regression estimate for both quotients.

Finally, it appears that maternal schooling had a significant positive effect on GDQ and DQ for scales A to D, as shown in Table 3.

## 4. Discussion

The present study was undertaken with the primary objective of investigating the early global development in children after they start using a CI. The results suggested a beneficial effect of cochlear implant use on early development, as measured by scale B (language and communication) of the Griffiths III test. After one year of CI use, a significant increase in DQ was observed, which approached normal levels.

Scale B is intended to measure overall language development, including expressive language, receptive language, and the use of language to communicate socially with others.

After cochlear implantation, children can start to use linguistic sounds they perceive during everyday interactions to gradually develop their language system. This is fundamental for understanding and giving sense to the reality around them. Language makes it possible to communicate about objects, events, or ideas, even if they are not witnessed directly, such as past events or faraway places [21]. It can also help children to take different perspectives on the same events and to grasp the point of view of others regarding objects and situations [22].

Also, Pantelemon et al. [23] assessed general and linguistic development before and after CI (3 and 6 months of follow-up) in a cohort of 17 children with a mean age at CI implantation of 22 months. The development was evaluated using the Denver Developmental Screening test (DDST II), a test battery designed for children aged 0 to 6 years. This test evaluates various aspects of a child’s development including personal–social, fine motor-adaptive skills, language skills, and gross motor skills. In accordance with our results, they identified statistically significant differences between language DQ evaluations conducted before CI and 3 months and 6 months later. Unlike our results, they also found significant differences in overall DQ throughout their early short-term follow-up. It is possible that the observed differences can be explained by methodological differences between these studies. It is worth noting that our sample includes a population that received their implant earlier (at 14 months) and was reassessed later (at 12 months after CI). We can conclude from our data that a positive effect of a CI on language development is evident very early on and is maintained even one year after beginning CI use.

It was unexpected for us to find no significant differences in the other scales between t0 and t1, particularly in the GDQ. The explanation could be the period between t0 and t1 (12 months) being too short to notice changes in other cognitive domains. Other authors [24] who used the first version of the Griffiths test found significant changes in the locomotor, learning, and eye–hand coordination scales five years after cochlear implant activation. Deafness undoubtedly has consequences on brain development beyond hearing impairment, even after cochlear implantation [25]: it can be described as a connectome disease, in which impaired links are found not only within the auditory system, but also in other sensory systems. This has severe negative effects on high-level cognitive functions. In particular, for prelingually deaf people who receive a cochlear implant at an early age, at least three related areas of neurocognitive functioning are at risk for delayed or atypical development: executive functioning; sequential processing and sequence learning; and concept formation. It is essential to investigate the effects of CI use on other Griffiths III scales after a long period of CI use.

Our secondary aim was to investigate the role of other variables in early development. These variables include age at CI activation, gender, and parental schooling.

We found a significant negative correlation between the age at CI activation and both DQ score at scale B and the GDQ.

These findings support the hypothesis that CI not only restores hearing but also contributes to the broader cognitive architecture necessary for developmental progression. Early auditory stimulation through CI enhances neural plasticity, supporting more efficient development of both receptive and expressive language skills [5,26,27,28].

This result is expected, as many studies have shown the effectiveness of CI on children’s general and linguistic development [29,30,31,32,33].

Kronenberger and Pisoni [34] demonstrated that children with early CI activation outperform peers who received their implants later in neurocognitive domains, particularly verbal working memory, which is crucial for language development outcomes.

Colletti et al. [24] also found a positive effect of early intervention on the cognitive development of CI children. Using the Griffiths Mental Developmental Scale (GMDS), they compared the performances at 5 years of CI follow-up of children who received the implant before 12 months of age with those of children who received surgery between 13 and 23 months and 24 and 35 months of age. They demonstrated improved cognitive performance in children who received their implant before 12 months of age compared to those who received the implant later. Early restoration of auditory input through CI allows children to benefit from auditory verbal and non-verbal stimuli. This helps them master more complex skills in a very ‘‘critical period’’ of their development and to take maximum advantage of brain plasticity. This prevents degenerative alterations and facilitates appropriate functional development within the brain [10].

No significant correlations were found between gender and early global development, contradicting our initial assumptions. It is well known that the gender variable has a significant effect on a child’s language development. Girls are demonstrably ahead of boys in early communicative gestures, in productive vocabulary, and in combining words [35]. Girls talk earlier and more because they have more and different types of language stimulation [5]. We did not find any differences, possibly because a larger sample is needed.

The children’s paternal education level did not have a significant influence on their early global development. Few studies have directly examined the impact of fathers on deaf children’s outcomes [36]. The presence of a father in the lives of deaf children has been shown to positively impact children’s academic and language development [37]. One might expect that fathers’ involvement in early intervention would have a positive effect on children’s outcomes. For example, fathers who were more involved in early intervention showed higher levels of self-efficacy and perceived more social support from their networks than less-involved fathers did. These involved fathers were described as having a better understanding of their children and as finding joy in their involvement with them [38]. The lack of significance of paternal education in our study could be attributed both to the size of our sample and probably to cultural aspects. Currently, in Italy, it is still mainly women who take care of their children’s upbringing. However, a change is beginning to take place, with fathers also becoming more involved. It would be interesting to investigate these same factors further down the line.

Finally, a significant correlation was found between the mother’s schooling and the DQ scores on all scales; the higher the mother’s schooling, the higher the DQ scores. Our findings align with the literature demonstrating that higher maternal education levels are associated with richer language input and more cognitively stimulating environments [10]. Properties of maternal speech, directly related to the education level of the mother, determine different rates of productive vocabulary development in children. In fact, higher education implied that mothers used a greater number and more types of words, sentences with multiple subordinate clauses, and more affirmative feedback [39].

Furthermore, the mother’s education level is directly correlated with her active involvement in the child’s education and in the post-surgery rehabilitation process. Socioeconomic factors, including household income and parental education level, are also important prognostic factors for children with normal hearing to develop communication skills as cultural and intellectual activities within the family are involved in children’s development and positively affect psycho-emotional and language development [40].

These factors have a significant impact on a child’s vocabulary growth, pragmatic skills, and social–emotional development, as confirmed by recent longitudinal studies [30,33].

Furthermore, evidence suggests that maternal responsiveness, narrative elaboration, and joint attention activities positively affect not only a child’s linguistic abilities but also, as we found, cognitive growth [41].

Early intervention programs that actively involve parents, especially mothers, in rehabilitation have been shown to enhance outcomes in children with CI. Children from families involved in the verbal rehabilitation process show significant improvements in language skills [3,4].

## 5. Conclusions

After one year of CI use, a significant improvement of the early global development of children was found in the language and communication domain.

The age at CI activation and the level of the mother’s education were found to be related to early global development.

Our findings reinforce the need for a multidisciplinary approach that combines audiological rehabilitation with neurocognitive monitoring and parental training, especially in the early years of development when neuroplasticity is at its peak.

## 6. Limitations

This study has several limitations that should be acknowledged. First, the relatively small sample size reduces statistical power and may limit the generalizability of the findings. Additionally, the short follow-up period may not capture long-term developmental outcomes or changes over time adequately. The absence of a control group hinders direct comparisons with children who did not receive cochlear implants or who underwent alternative interventions, thereby restricting causal interpretations. Finally, despite efforts to ensure consistency and reliability in data collection, potential observer bias may have influenced the outcome assessments, particularly in measures involving subjective interpretation. These limitations underscore the necessity of further research involving larger cohorts, longer follow-up periods, and controlled designs to validate and expand upon the current findings.

## Figures and Tables

**Table 1:** Sample characteristics.

n. Subjects	Gender	Median Age at Diagnosis (Months)	Median Age at CI Activation (Months)	Mean Age at t0 (Months)	Mean Age at t1 (Months)	Mother’s Schooling Mean Age (Years)	Father’s Schooling Mean Age (Years)
24	12 females 12 males	3	14	13.7	24.4	15.2	14

**Table 2:** Development quotients (DQs) at different Griffiths III scales and the general development quotient (GDQ) before (t0) and after (t1) cochlear implantation. The displayed *p*. values are corrected for multiplicity using a Benjamini–Hochberg correction.

	t0 Mean (sd)	t1 Mean (sd)	Test Statistic	*p*. Value
Scale A (DQ)	98.3 (19.7)	99.04 (11.5)	−0.23575	0.816
Scale B (DQ)	63.6 (16.2)	78.9 (19.8)	−2.76644	0.011
Scale C (DQ)	98.1 (16.1)	93.2 (15)	1.408743	0.172
Scale D (DQ)	98 (13.2)	92.6 (15.2)	1.685367	0.105
Scale E (DQ)	96.7 (17.1)	96.6 (14.6)	0.041407	0.967
GDQ	84.5 (16.3)	87.4 (18.1)	−0.99263	0.331

**Table 3:** Regression coefficient associated with mother’s schooling (in years) in multivariate regression for DQ and GDQ scores.

Response	Estimate	std. Error	Statistic	*p*. Value
Scale A_DQ	2.433852	0.908866	2.6779	0.013 *
Scale B_DQ	2.038208	0.966431	2.10901	0.045 *
Scale C_DQ	2.348758	0.842754	2.797	0.009 **
Scale D_DQ	1.914092	0.764038	2.50523	0.019 *
Scale E_DQ	1.758525	0.99993	1.75865	0.091 (.)
GDQ	2.202218	0.986047	2.23338	0.035 *

Signif. codes: 0.001 ** 0.01 * 0.05 (.) 0.1 ‘ ’ 1.

## Data Availability

The data presented in this study are available on request from the corresponding author due to privacy restrictions.

## Abbreviations

The following abbreviations are used in this manuscript:

CI	Cochlear Implant
CIs	Cochlear Implants
HL	Hearing Loss
PTA	Pure Tone Average
HAs	Hearing Aids
DQ	Development Quotient
GDQ	Global Development Quotient

## References

[1] Yoshinaga-Itano C., Sedey A.L., Wiggin M., Mason C.A. (2018). Language outcomes improved through early hearing detection and earlier cochlear implantation. Otol. Neurotol..

[2] Nicholas J.G., Geers A.E. (2007). Will they catch up? The role of age at cochlear implantation in the spoken language development of children with severe to profound hearing loss. J. Speech Lang. Hear. Res..

[3] Geers A.E., Brenner C., Tobey E.A. (2011). Long-Term outcomes of cochlear implantation in early childhood: Sample characteristics and data collection methods. Ear Hear.

[4] Niparko J.K., Tobey E.A., Thal D.J., Eisenberg L.S., Wang N.Y., Quittner A.L., Fink N.E. (2010). Spoken language development in children following cochlear implantation. JAMA.

[5] Guerzoni L., Mancini P., Nicastri M., Fabrizi E., Giallini I., Cuda D. (2020). Does early cochlear implantation promote better reading comprehension skills?. Int. J. Pediatr. Otorhinolaryngol..

[6] Guerzoni L., Falzone C., Ghiselli S., Nicastri M., Mancini P., Fabrizi E., Cuda D. (2025). Corrigendum to “Speech Perception in noise in adolescents with cochlear implant”. Int. J. Pediatric Otorhinolaryngol..

[7] Mancini P., Nicastri M., Giallini I., Odabaşi Y., Greco A., D’Alessandro H.D., Portanova G., Mariani L. (2023). Long-term speech perception and morphosyntactic outcomes in adolescents and young adults implanted in childhood. Int. J. Pediatr. Otorhinolaryngol..

[8] Geers A.E. (2004). Speech, language, and reading skills after early cochlear implantation. Arch. Otolaryngol. Head Neck Surg..

[9] DesJardin J.L., Eisenberg L.S. (2007). Maternal contributions: Supporting language development in young children with cochlear implants. Ear Hear..

[10] Kral A., Yusuf P.A., Land R. (2017). Higher-order auditory areas in congenital deafness: Top-down interactions and corticocortical decoupling. Hear Res..

[11] Harris M.S., Hamel B.L., Wichert K., Kozlowski K., Mleziva S., Ray C., Pisoni D.B., Kronenberger W.G., Moberly A.C. (2023). Contribution of Verbal Learning & Memory and Spectro-Temporal Discrimination to Speech Recognition in Cochlear Implant Users. Laryngoscope.

[12] Porto L., Wouters J., van Wieringen A. (2023). Speech perception in noise, working memory, and attention in children: A scoping review. Hear Res..

[13] McCreery R.W., Walker E.A., Spratford M., Lewis D., Brennan M. (2019). Auditory, Cognitive, and Linguistic Factors Predict Speech Recognition in Adverse Listening Conditions for Children with Hearing Loss. Front. Neurosci..

[14] Almomani F., Al-Momani M.O., Garadat S., Alqudah S., Kassab M., Hamadneh S., Rauterkus R., Gans R. (2021). Cognitive functioning in Deaf children using Cochlear implants. BMC Pediatrics.

[15] Hashemi S.B., Monshizadeh L. (2012). The effect of cochlear implantation in development of intelligence quotient of 6–9 deaf children in comparison with normal hearing children (Iran, 2009–2011). Int. J. Pediatr. Otorhinolaryngol..

[16] Fei P., Shehata-Dieler W., Huestegge L., Hagen R., Kühn H. (2023). Longitudinal Development of Verbal and Nonverbal Intelligence After Cochlear Implantation According to Wechsler Tests in German-speaking Children: A Preliminary Study. Ear Hear..

[17] The Association for Research in Infant and Child Development (2018). Griffiths III Year Four Items Grouped by Quartile Level of Difficulty. https://www.aricd.ac.uk/.

[18] Lanfranchi S., Rea M., Ferri R., Vianello R. (2019). Studio di Validazione e Standardizzazione Italiana delle Griffiths III.

[19] Bates D., Maechler M., Bolker B., Walker S. (2015). Fitting Linear Mixed-Effects Models Using lme4. J. Stat. Softw..

[20] R Core Team (2023). R: A Language and Environment for Statistical Computing. R Foundation for Statistical Computing, Vienna, Austria. https://www.R-project.org/.

[21] Luchkina E., Waxman S. (2024). Talking About the Absent and the Abstract: Referential Communication in Language and Gesture. Perspect. Psychol. Sci..

[22] Kaltefleiter L.J., Sodian B., Kristen-Antonow S., Grosse Wiesmann C., Schuwerk T. (2021). Does syntax play a role in Theory of Mind development before the age of 3 years?. Infant Behav. Dev..

[23] Pantelemon C., Necula V., Berghe A.S., Livinț-Popa L., Palade S., Văcăraș V., Mureșanu I.A., Strilciuc S., Mureșanu F.D. (2020). Neurodevelopmental Aspects and Cortical Auditory Maturation in Children with Cochlear Implants. Medicina.

[24] Colletti L., Mandalà M., Zoccante L., Shannon R.V., Colletti V. (2011). Infants versus older children fitted with cochlear implants: Performance over 10 years. Int. J. Pediatr. Otorhinolaryngol..

[25] Kral A., Kronenberger W.G., Pison D.B., O’Donoghue G.M. (2016). Neurocognitive factors in sensory restoration of early deafness: A connectome model. Lancet Neurol..

[26] Svirsky M.A., Teoh S.W., Neuburger H. (2004). Development of language and speech perception in congenitally, profoundly deaf children as a function of age at cochlear implantation. Audiol. Neuro-Otol..

[27] Tomblin J.B., Peng S.C., Spencer L.J., Lu N. (2008). Long-term trajectories of the development of speech sound production in pediatric cochlear implant recipients. J. Speech Lang. Hear. Res..

[28] Spencer L.J., Tomblin B. (2009). Evaluating phonological processing skills in children with prelingual deafness who use cochlear implants. J. Deaf Stud. Deaf Educ..

[29] Kral A., Sharma A. (2019). Developmental neuroplasticity after cochlear implantation. Trends Neurosci..

[30] Ambrose S.E., Walker E.A., Unflat-Berry L.M., Oleson J.J., Moeller M.P. (2020). Quantity and quality of caregivers’ linguistic input to 18-month and 3-year-old children who are hard of hearing. Ear Hear..

[31] Cejas I., Mitchell C.M., Barker D.H., Sarangoulis C., Eisenberg L.S., Quittner A.L. (2021). Parenting Stress, Self-Efficacy, and Involvement: Effects on Spoken Language Ability Three Years After Cochlear Implantation. Otol. Neurotol..

[32] Mueller L., Adkins D., Kao A., Munyemana M.A., Kallogjeri D., Lieu J.E. (2025). Social Determinants of Health and Language and Academic Outcomes in Pediatric Cochlear Implantation: A Systematic Review and Meta-Analysis. JAMA Otolaryngol. Head Neck Surg..

[33] Nittrouer S. (2021). The changing face of spoken language development in children with hearing loss. J. Deaf Stud. Deaf Educ..

[34] Kronenberger W.G., Pisoni D.B. (2020). Neurocognitive functioning in deaf children with cochlear implants. Curr. Opin. Otolaryngol. Head Neck Surg..

[35] Davidson L.S., Geers A.E., Blamey P.J., Tobey E.A., Brenner C.A. (2011). Factors contributing to speech perception scores in long-term pediatric cochlear implant users. Ear Hear.

[36] Szarkowski A., Dirks E. (2021). Fathers of Young Deaf or Hard-of-Hearing Children: A Systematic Review. J. Deaf Stud. Deaf Educ..

[37] Calderon R., Low S. (1998). Early social-emotional, language and academic development in children with hearing loss: Families with and without fathers. Am. Ann. Deaf.

[38] Ingber S., Most T. (2012). Fathers’ involvement in preschool programs for children with and without hearing loss. Am. Ann. Deaf.

[39] Majorano M., Guidotti L., Guerzoni L., Murri A., Morelli M., Cuda D., Lavelli M. (2018). Spontaneous language production of Italian children with cochlear implants and their mothers in two interactive contexts. Int. J. Lang. Commun. Disord..

[40] Lee C., Son S.E., Moon I.J., Chung W.H., Cho Y.S., Hong S.H., Cho Y.S. (2025). The role of socioeconomic factors and third-party support in language development for children with cochlear implants. Sci. Rep..

[41] Meinzen-Derr J., Wiley S., Grether S., Choo D.I., Murray J. (2022). Home intervention and early language outcomes in young children with hearing loss. Pediatrics.

